# Gastric cancer-derived mesenchymal stromal cells trigger M2 macrophage polarization that promotes metastasis and EMT in gastric cancer

**DOI:** 10.1038/s41419-019-2131-y

**Published:** 2019-12-04

**Authors:** Wei Li, Xu Zhang, Fenglei Wu, Ying Zhou, Zengtao Bao, Haining Li, Ping Zheng, Shaolin Zhao

**Affiliations:** 1 Center of Research Laboratory, The First People’s Hospital of Lianyungang, Lianyungang, 222001 China; 2 Department of Pathology, Xuzhou Medical University, Xuzhou, 221004 China; 3 Jiangsu Key Laboratory of Medical Science and Laboratory Medicine, Jiangsu University, Zhenjiang, 212013 China; 4 Department of Oncology, The First People’s Hospital of Lianyungang, Lianyungang, 222001 China; 5 Department of Gastrointestinal Surgery, The First People’s Hospital of Lianyungang, Lianyungang, 222001 China; 6 Department of Clinical Laboratory Diagnostics, Kangda College of Nanjing Medical University, Lianyungang, 222000 China

**Keywords:** Cancer microenvironment, Gastric cancer

## Abstract

Resident macrophages in the tumor microenvironment exert a dual role in tumor progression. So far, the mechanism of intratumoral macrophage generation is still largely unknown. In the present study, the importance of macrophages in the pro-tumor role of gastric cancer-derived mesenchymal stromal cells (GC-MSCs) was observed in a mouse xenograft model with macrophage depletion. In gastric cancer tissues, high expression levels of Ym-1, Fizz-1, arginase-1, and CCR-2, as well as a low expression level of iNOS, were verified, and co-localization of GC-MSCs and tumor-associated macrophages (TAMs) was observed by dual immunofluorescence histochemistry. TAMs isolated from gastric cancer tissues predominantly displayed an M2 phenotype. In a co-culture system, the contribution of GC-MSCs to M2 polarization of macrophages was confirmed by the M2-related protein expression, M2-like immunophenotype and cytokine profile of GC-MSC-primed macrophages in vitro. Blockade of IL-6/IL-8 by neutralizing antibodies significantly attenuated the promoting effect of GC-MSCs on M2-like macrophage polarization via the JAK2/STAT3 signaling pathway. In addition, GC-MSC-primed macrophages promoted the migration and invasion of gastric cancer cells, and the process of EMT in gastric cancer cells was significantly enhanced by GC-MSC-primed macrophage treatment. Our study showed that tumor-promoting GC-MSCs contribute to M2 macrophage polarization within the gastric cancer niche through considerable secretion of IL-6 and IL-8. These GC-MSC-primed macrophages can subsequently prompt gastric cancer metastasis via EMT promotion in gastric cancer cells.

## Introduction

Gastric cancer is the second most prevalent malignant tumors worldwide^1^. Although advancement in treatment has resulted in a decrease in mortality, the 5-year survival rate of patients after surgical resection remains low^2^. Among the causes, a high rate of metastasis is a major obstacle in improving long-term survival after curative resection^3^. Therefore, elucidation of a unique pathological mechanism for gastric cancer progression is urgently needed to search for effective therapeutic targets for gastric cancer. Accumulating evidence suggests that macrophages are critical for the occurrence and development of various tumors^4,5^. A high rate of macrophage infiltration is associated with poor prognosis of gastric cancer and the markers of macrophages can be used as prognostic indexes^6^.

In diverse tumor stroma, tumor-associated macrophages (TAMs) have high plasticity and predominantly present as an M2 phenotype, which is associated with cancer metastasis and worse prognosis in patients^7,8^. However, the underlying mechanism for M2-like TAM generation within the tumor microenvironment is largely unknown. The effects of cancer cells on the M2 polarization of macrophages have become a research focus. Factors secreted by cervical cancer cells have been demonstrated to induce a stable M2 phenotypic profile in THP-1-derived macrophages in vitro^9^. Breast cancer-derived hyaluronan also stimulates the formation of M2-like TAMs in a mouse model^10^. Peritoneal TAMs are also polarized to an M2 phenotype by gastric cancer cells^11^. Nevertheless, the contribution of tumor stromal cells to the generation of M2-like TAMs is still obscure and could be critical for identifying better potential therapeutic targets in gastric cancer.

Tumor stromal cells have been reported to exert an important role in the initiation and progression of various tumors^12,13^. As one of the key components in tumor stroma, mesenchymal stromal cells (MSCs) have attracted much attention and their unique effect on tumorigenesis and progression has been noted^14,15^. Within the tumor microenvironment, MSCs exert their immunosuppressive potential via interacting with a variety of immune cells, such as lymphocytes, dendritic cells and macrophages^16,17^. One of our previous studies indicated that human umbilical cord-derived stromal cells can notably promote M2 polarization of macrophages at sites of renal ischemia-reperfusion injury^18^. Following that, in another study, we isolated cancer-associated MSCs from gastric cancer tissues, named as gastric cancer-derived MSCs (GC-MSCs), and found their ability to promote the proliferation, migration and angiogenesis of gastric cancer cells; they are more potent than non-cancerous tissue-derived MSCs^19^. Thereafter, we investigated the regulatory effect of GC-MSCs on M2-like TAM generation in gastric cancer and, subsequently, the role of GC-MSC-primed macrophages in prompting tumor progression.

In this study, we reported for the first time that the polarization and generation of pro-tumor M2-like macrophages was strikingly triggered by GC-MSCs in gastric cancer through activation of the JAK2/STAT3 signaling pathway via high secretion of IL-6/IL-8. Moreover, GC-MSC-primed macrophages remarkably promoted gastric cancer metastasis by enhancing the process of EMT in gastric cancer cells.

## Results

### Macrophages are essential for the tumor-promoting effect of GC-MSCs in vivo

**Fig. 1:**
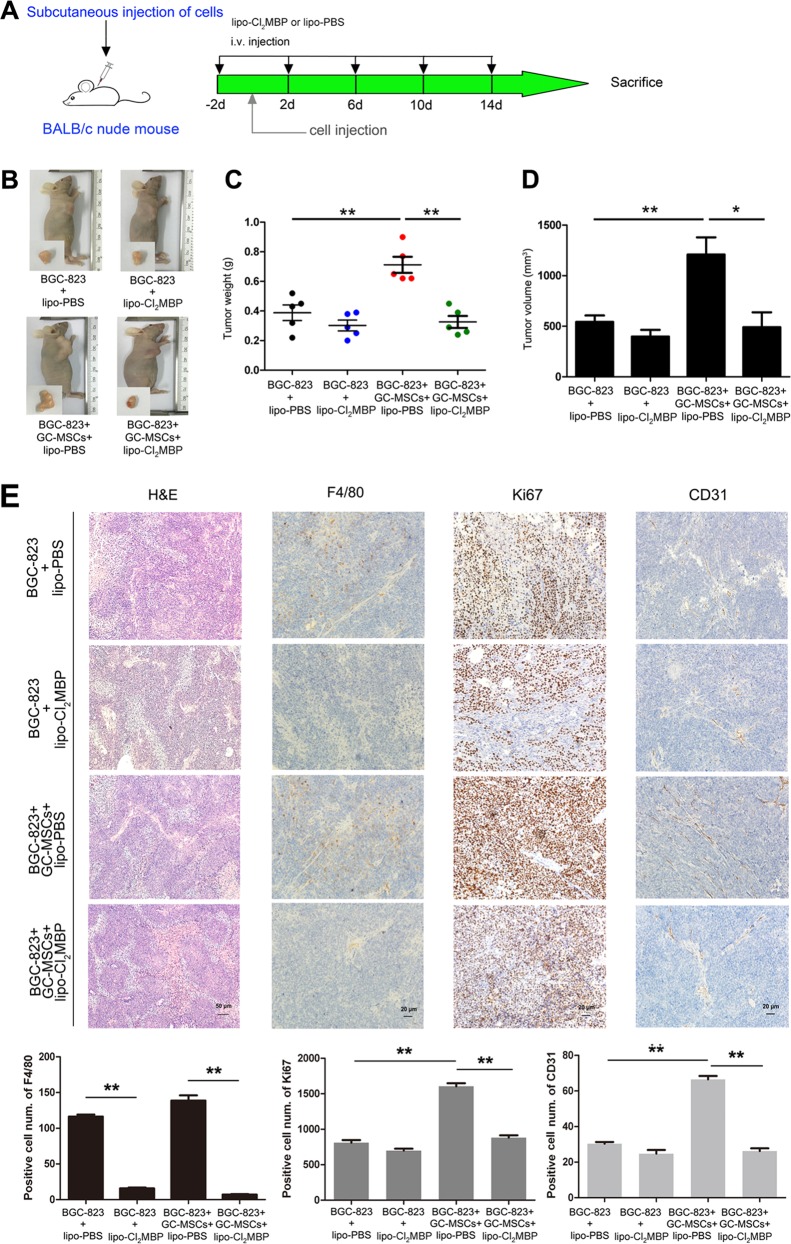
Macrophage-depletion dampens tumor growth facilitated by GC-MSCs in a mouse xenograft model. **a** Experimental design of macrophage-depletion by clodronate liposome application in gastric cancer-bearing BALB/c nude mice. **b** Representative gross morphology of tumor at 14 days after BGC-823 transplantation with distinct treatments. **c** Tumor weight and **d** tumor volume evaluated in each group of mice at 14 days after BGC-823 transplantation with distinct treatments. *n* = 5 in each group. **e** Representative histopathology images of tumors collected from mice co-injected by BGC-823 and GC-MSCs, with or without macrophage-depletion. H&E staining: scale bar, 50 µm; Immunohistochemistry for F4/80: scale bar, 20 µm; for Ki67: scale bar, 20 µm; for CD31: scale bar, 20 µm. *P < 0.05; **P < 0.01.

GC-MSCs were characterized by their pluripotent differentiation potential and immunophenotype. After 7–14 days of primary culture, the cells emerged with fibroblast-like morphology, with both osteoblastic and adipogenic differentiation potential, and the cells were positive for CD29, CD44, CD90, and CD105 but negative for CD14, CD34, and CD45 (Supplementary Fig. 1). To assess the contribution of macrophages to the pro-tumor effect of GC-MSCs, we performed tumor xenograft experiments in BALB/c nude (nu/nu) mice with concomitant depletion of monocytes/macrophages by lipo-Cl_2_MBP, which is a macrophage ‘suicide’ agent developed by van Rooijen et al.^20^. As shown in Fig. 1a, monocytes/macrophages were depleted in each cohort of tumor-bearing mice by lipo-Cl_2_MBP application via intravenous injection at different time points during the experimentation period. After subcutaneous co-injection with GC-MSCs at a 5:1 ratio for 14 days, BGC-823-implanted mice displayed significant increases in either tumor weight or volume compared with the control cohort of mice (Fig. 1b–d). However, macrophage depletion strikingly abrogated the tumor-promoting effect of GC-MSCs (Fig. 1b–d). In agreement with this, a more severe histopathology was observed by H&E staining in the cohort of mice with BGC-823 and GC-MSC co-injection than in the control cohort, and the severity of malignant tumor significantly decreased upon macrophage depletion (Fig. 1e). The efficiency of myeloid cell depletion by lipo-Cl_2_MBP was confirmed by analyzing tumor tissues for F4/80^+^ cell infiltration (Fig. 1e). The results of Ki67 and CD31 staining indicated increased intratumor proliferation and angiogenesis, respectively, in the cohort of mice with co-injection compared with the control cohort, which was also abrogated by macrophage depletion (Fig. 1e). In another mouse xenograft model with MKN-28, similar results were also observed (Supplementary Fig. 2). Thus, our results indicate a key role of macrophages in the potent pro-tumor effect of GC-MSCs. The properties of TAMs and the potential contribution of GC-MSCs to TAM generation within gastric cancer drew our attention.

### TAMs in gastric cancer with GC-MSC co-existence predominantly present with the M2 subtype

**Fig. 2:**
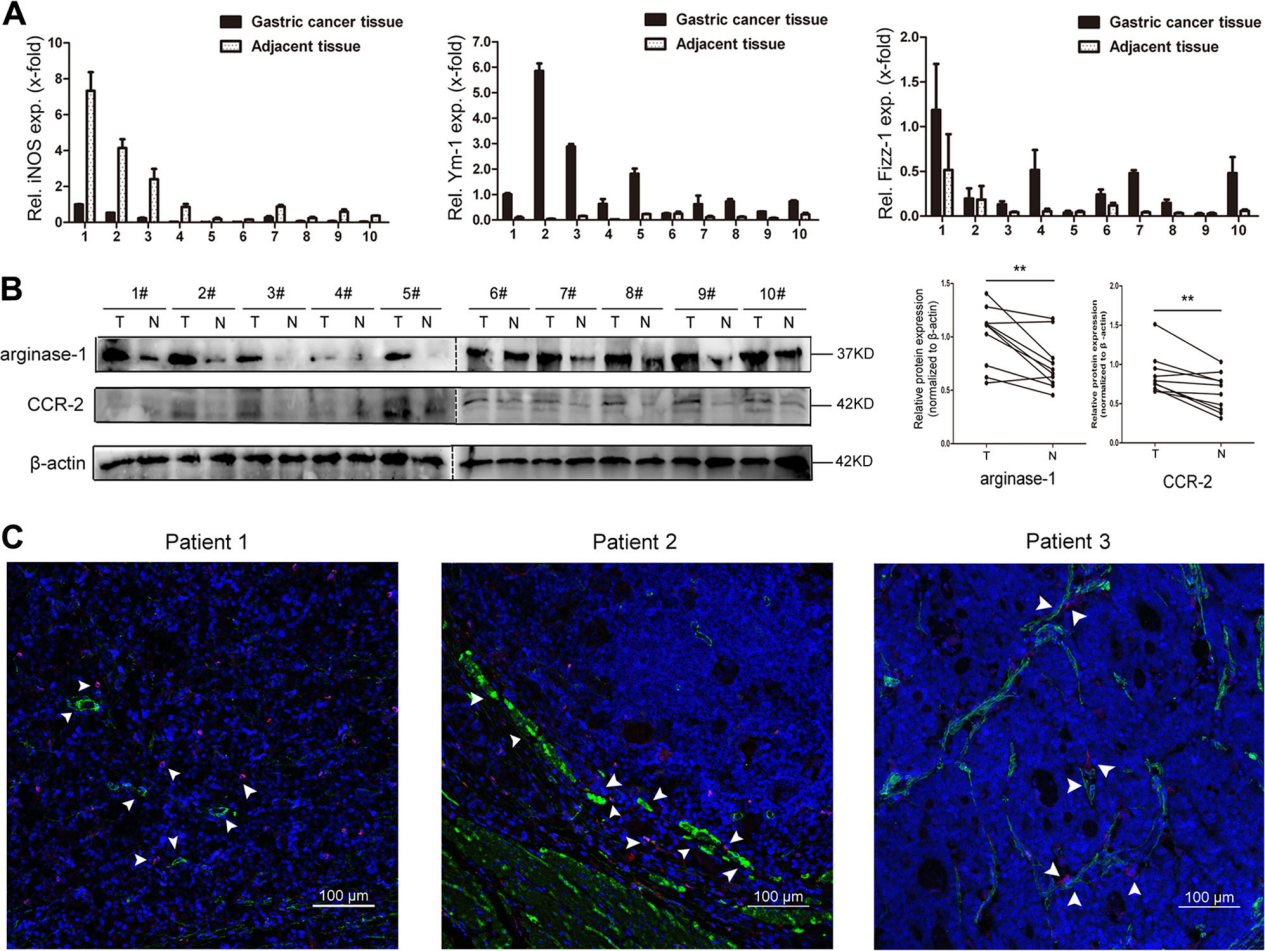
M2 macrophage-related protein expression and the co-location of M2-macrophages and GC-MSCs within tumor tissues. **a** Expression levels of iNOS, Ym-1, and Fizz-1 mRNA were analyzed by RT-qPCR in tumor tissues and the corresponding adjacent non-cancerous tissues collected randomly from 10 gastric cancer patients. **b** The expressions of arginase-1 and CCR-2 were assessed by western blot in tumor tissues and the corresponding adjacent non-cancerous tissues collected randomly from 10 gastric cancer patients. **c** Representative immumofluoresence images of the co-location of GC-MSCs (α-SMA; green) and M2-macrophages (CD204; red) in gastric cancer tissues detected by confocal microscopy. scale bar, 100 µm. **P < 0.01 vs. gastric cancer tissue. T: tumor; N: non-cancerous tissues.

To study the potential correlation between GC-MSCs and macrophages, we expanded our observation to include tumor tissues from 10 randomly selected clinical gastric cancer patients. Compared with the adjacent non-cancerous tissues, significantly higher levels of Ym-1, Fizz-1, arginase-1 and CCR-2, the M2 macrophages’ markers, were observed in gastric cancer tissues with GC-MSCs infiltration (Fig. 2a, b). We also collected tumor tissues from gastric cancer cell-implanted mice at 14 days. Compared with the control cohort, the expression level of iNOS mRNA was significantly lower in the experimental cohort with GC-MSC co-injection, whereas Ym-1, Fizz-1 and arginase-1 were all highly expressed in the co-injection cohort (Supplementary Fig. 3). In addition, the confocal immunofluorescence images displayed an obvious co-localization of GC-MSCs and M2-type macrophages within tumor tissues collected from three representative gastric cancer patients (Fig. 2c).

**Fig. 3:**
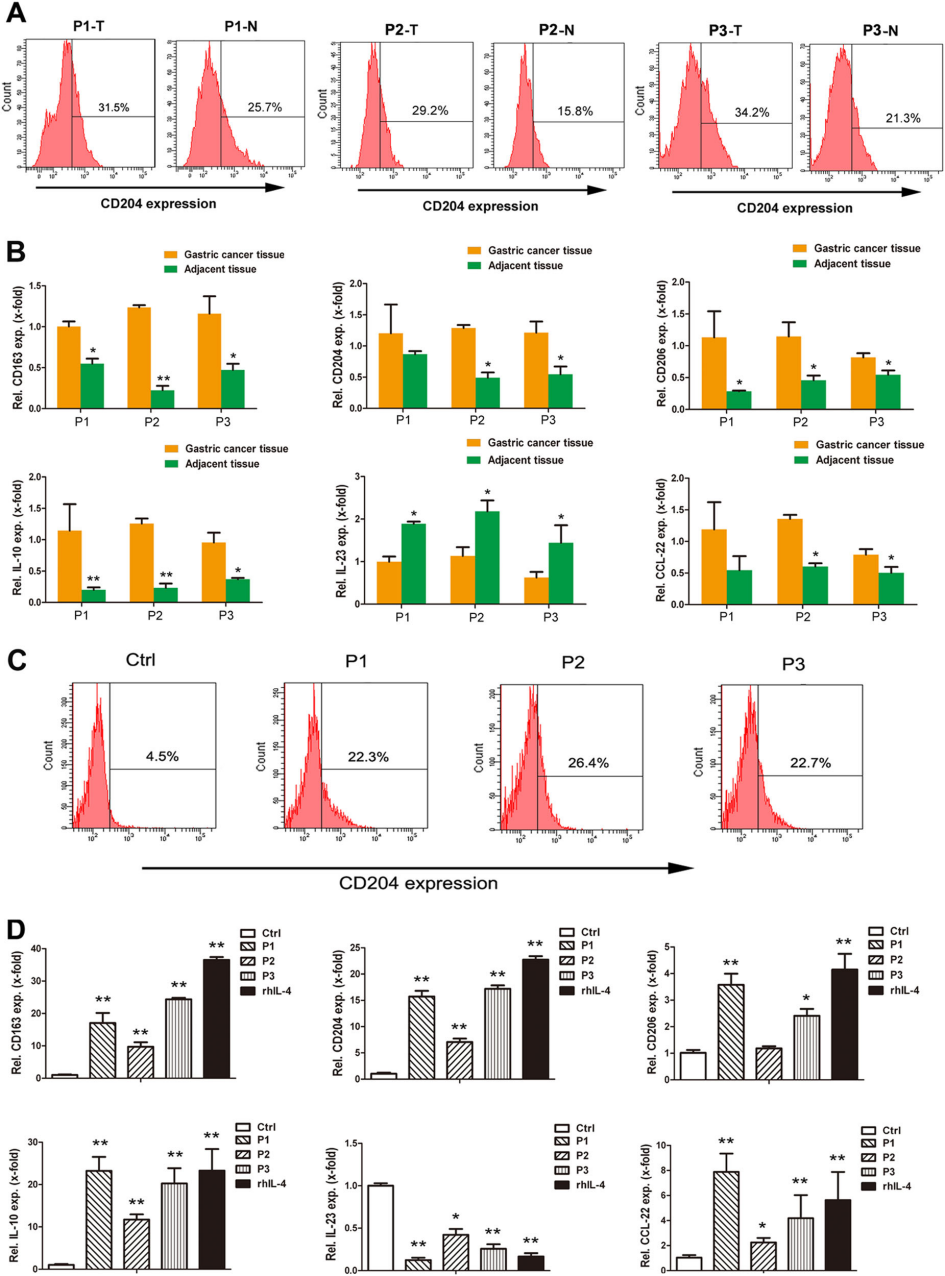
Macrophages picked up from tumor tissue or peripheral blood of gastric cancer patients present as a M2-like phenotype prominently. **a** The proportion of CD204^+^ cells was detected by flow cytometry in macrophages harvested by MACS from tumor tissues and the corresponding adjacent non-cancerous tissues of three representative gastric cancer patients. **b** Expression levels of CD163, CD204, CD206, IL-10, CCL-22, and IL-23 mRNA were detected by RT-qPCR in macrophages of tumor tissue and the corresponding adjacent non-cancerous tissue origin. **c** The proportion of CD204^+^ cells was detected by flow cytometry in macrophages of peripheral origin harvested by MACS from three representative gastric cancer patients and the healthy control. **d** Expression levels of CD163, CD204, CD206, IL-10, CCL-22, and IL-23 mRNA were detected by RT-qPCR in macrophages of peripheral origin. *P < 0.05; **P < 0.01 vs. Ctrl. T: tumor; N: non-cancerous tissues; Ctrl: control; *P*: patient.

Thereafter, the infiltrated macrophages were further sorted by magnetic cell sorting (MACS) in either the cancer tissues or the corresponding non-cancerous tissues. The flow cytometric assay displayed a higher ratio of CD204^+^ cells in macrophages derived from the human gastric cancer tissues than in macrophages derived from the corresponding non-cancerous tissues (Fig. 3a). RT-qPCR showed significantly higher levels of CD163, CD204, CD206, IL-10, and CCL-22 mRNA, which indicates an M2-like immunophenotype, in macrophages selected from the gastric cancer tissues than in those from the adjacent non-cancerous tissues, whereas the expression level of the M1-subtype marker IL-23 was significantly lower in gastric cancer tissue-derived macrophages (Fig. 3b). Next, we wondered whether the characterized phenotype of peripheral monocytes is different in gastric cancer patients and healthy controls, and the ratio of CD204^+^ cells was shown to be strikingly higher in macrophages collected from the peripheral blood of gastric cancer patients (Fig. 3c). RT-qPCR results also showed higher levels of CD163, CD204, CD206, IL-10, and CCL-22 mRNA in peripheral blood-derived macrophages selected from gastric cancer patients than from healthy controls (Fig. 3d). These observations suggest that GC-MSCs may play a role in M2 macrophage polarization in gastric cancer progression.

### GC-MSCs promote the polarization of macrophages into an M2-like subtype in vitro

**Fig. 4:**
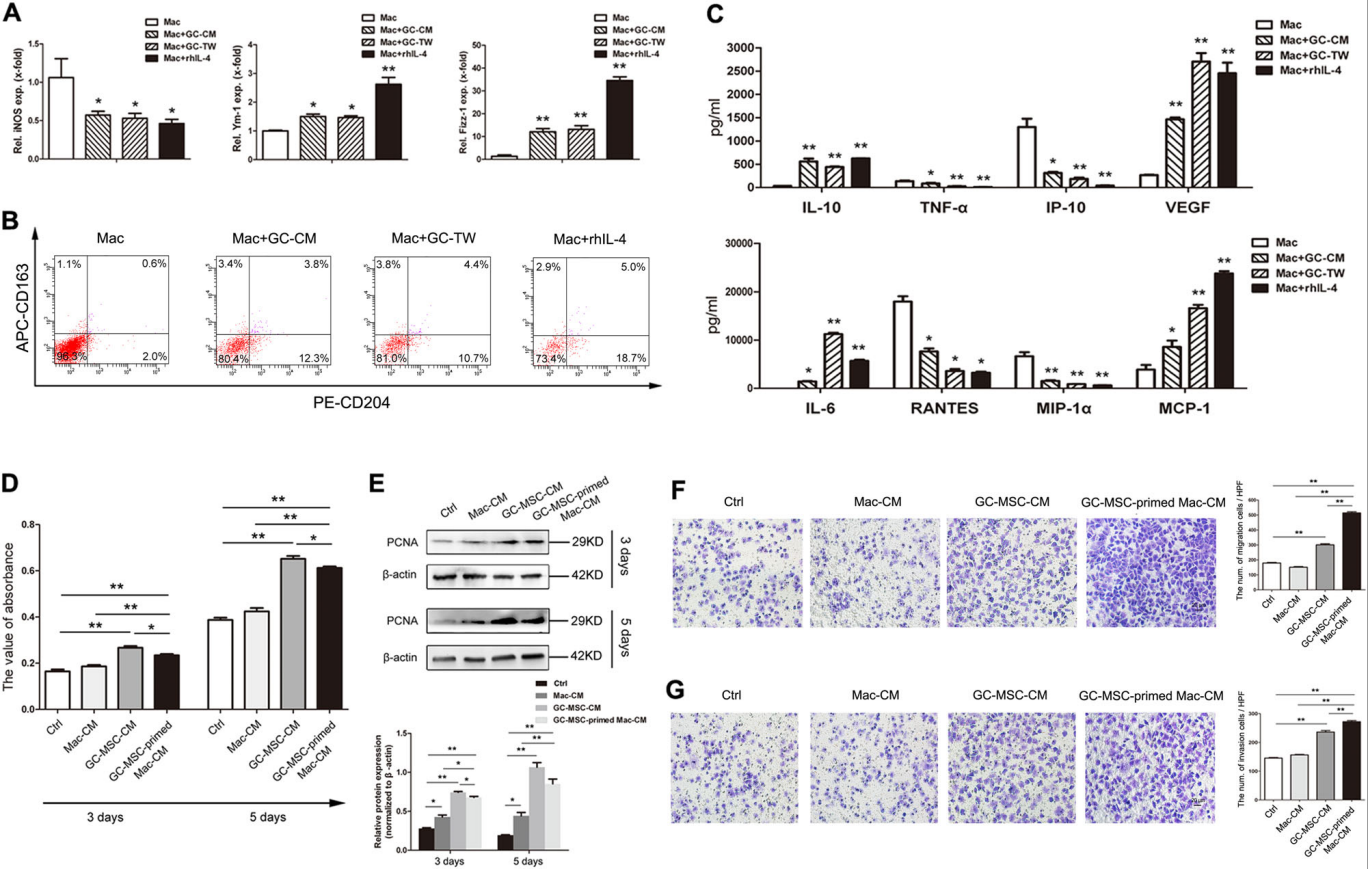
GC-MSCs promote the macrophage polarize into a M2-subtype, which facilitate the migration and invasion of gastric cancer cells potently. **a** The expression levels of iNOS, Ym-1, and Fizz-1 mRNA were detected by RT-qPCR in macrophages differentiated from human PBMCs co-cultured with GC-MSCs for 3 days. **b** The ratio of CD204^+^/CD163^+^ cells was analyzed by flow cytometry in macrophages after co-cultured for 3 days with GC-MSCs. **c** The supernatants of human PBMC-derived macrophages were collected at 3 days of co-culture with GC-MSCs and assessed by Luminex assay for the levels of cytokine profile. **d** The proliferation of MKN-28 cells was determined by using MTT assay after incubation with 20% CM from macrophages, GC-MSCs and GC-MSC-primed macrophages for 3 or 5 days. **e** The expression of PCNA in MKN-28 cells was analyzed by western blot after treatment by 20% CM from macrophages, GC-MSCs, and GC-MSC-primed macrophages. **f** The migration of MKN-28 cells was assessed in a transwell system with cells seeded in the upper charmer and 20% CM from macrophages, GC-MSCs and GC-MSC-primed macrophages added in the lower charmer. **g** The invasion of MKN-28 cells was analyzed in a Matrigel-coated transwell system. Scale bar, 20 µm. *P < 0.05; **P < 0.01. Ctrl: control; CM: conditioned medium; GC: gastric cancer; Mac: macrophages; TW: transwell.

To confirm whether M2 macrophage polarization is triggered by GC-MSCs, a co-culture system including GC-MSCs and human peripheral blood monocyte-derived macrophages was further exploited in our study. RT-qPCR results demonstrated higher expression levels of representative M2 macrophage markers in macrophages after co-culture with GC-MSCs for 3 days (Fig. 4a). Flow cytometric assays showed that GC-MSC treatment significantly increased the ratio of CD204^+^/CD163^+^ cells in macrophages at 3 days (Fig. 4b). In addition, by Luminex assay analysis, the concentrations of pro-inflammatory cytokines TNF-α, IP-10, RANTES, and MIP-1α significantly decreased in the supernatant of macrophages co-cultured with GC-MSCs, whereas the secretions of IL-6, IL-10, VEGF, and MCP-1, which are in favor of tumor progression, increased notably (Fig. 4c). Therefore, we demonstrated that GC-MSCs, one of the key pro-tumor stromal cell types, may play a potent role in inducing the M2 macrophage polarization within the gastric cancer microenvironment.

### GC-MSC-primed macrophages promote gastric cancer metastasis more potently than GC-MSCs do

One of our previous reports conveyed that GC-MSCs strikingly promote the proliferation and migration of gastric cancer cells, and this promotion is more potent than that in GCN-MSCs or bone marrow-derived MSCs (BM-MSCs)^19^. In this study, we observed that macrophages play an essential role in the tumor-promoting effect of GC-MSCs, and that GC-MSCs could in turn potently prompt the conversion of macrophages into the tumor-supporting M2 subtype. Thus, we investigated the potential roles of GC-MSC-primed macrophages in gastric cancer process and progression. As shown in Fig. 4d, CM from GC-MSC-primed macrophages significantly enhanced the proliferation of gastric cancer MKN-28 cells at either 3 days or 5 days of co-culture. However, the tumor-promoting ability of GC-MSC-primed macrophage-CM was not more potent than that of GC-MSC-CM (Fig. 4d). Similar results regarding the proliferative ability of MKN-28 cells with diverse treatments were obtained when examining PCNA expression by western blot (Fig. 4e). Transwell migration assays indicated that the number of migrated MKN-28 cells strikingly increased with GC-MSC-primed macrophage-CM, which was even more potent than with GC-MSC-CM (Fig. 4f). MKN-28 cells treated with GC-MSC-primed macrophage-CM also showed the highest cell invasion ability (Fig. 4g). Thus, GC-MSCs can regulate the M2 macrophage polarization within the tumor microenvironment, which in turn significantly promotes gastric cancer metastasis.

### IL-6 and IL-8 play important roles in GC-MSC-mediated macrophage polarization

**Fig. 5:**
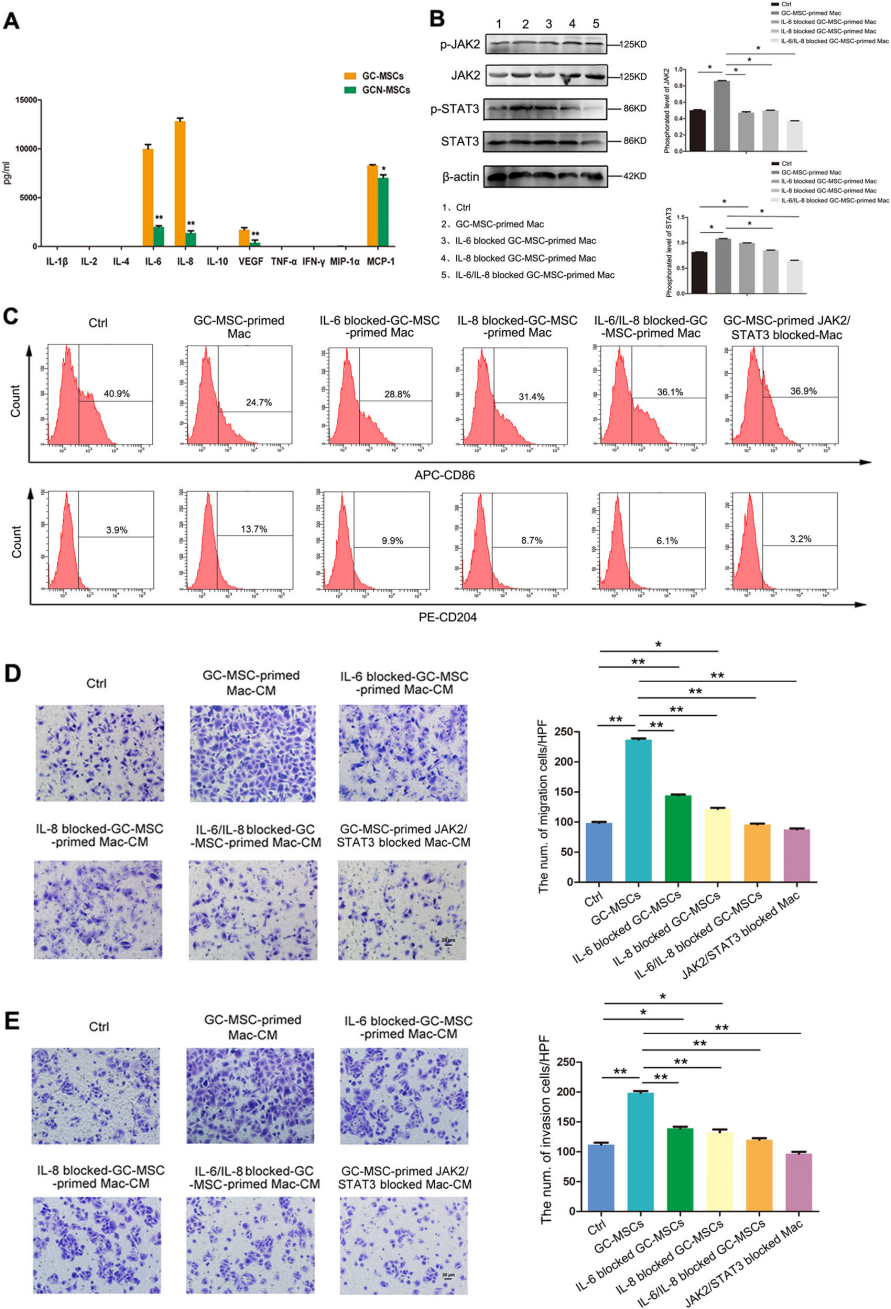
Regulating-effect of GC-MSCs on the M2-polarization of macrophages was partly mediated by IL-6 and IL-8 via the JAK2/STAT3 signaling pathway. **a** Cytokine profile analysis of GC-MSCs and GCN-MSCs by Luminex immunoassay. **b** Protein levels of JAK2, p-JAK2, STAT3, and p-STAT3 were detected by western blot in macrophages cultured in 20% GC-MSC-CM and anti-IL6/IL-8 pretreated GC-MSC-CM. **c** The proportion of CD86^+^ (M1) or CD204^+^ (M2) cells was detected by flow cytometry in macrophages cultured in 20% GC-MSC-CM and anti-IL6/IL-8 pretreated GC-MSC-CM, or treated by selective JAK2/STAT3 inhibitor (JSI-124) before cultured in 20% GC-MSC-CM. **d** The migration of MKN-28 cells was assessed in a transwell system with 20% CM from GC-MSC-primed macrophages and IL-6/IL-8-blocked GC-MSC-primed macrophages, or treated by JSI-124 before cultured in 20% GC-MSC-CM. **e** The invasion of MKN-28 cells was analyzed in a Matrigel-coated transwell system. Scale bar, 20 µm. *P < 0.05; **P < 0.01. Ctrl: control; CM: conditioned medium; GC: gastric cancer; Mac: macrophages; TW: transwell.

In this study, we further investigated the mechanism of M2-like polarization of macrophages prompted by GC-MSCs, and the key factors were screened in the supernatant of GC-MSCs and GCN-MSCs. After analysis by Luminex assay, cytokines/chemokines including VEGF, MCP-1, IL-6, and IL-8 were detectable in the CM of GC-MSCs and GCN-MSCs, whereas GC-MSCs produced a strikingly higher level of IL-6 and IL-8 secretion with significant differences from GCN-MSCs (Fig. 5a). Since both IL-6 and IL-8 are reported to play a paramount role in macrophage polarization^21,22^, we herein investigated the contribution of IL-6, IL-8, or IL-6 and IL-8 together to the promotion of macrophage phenotype switching by GC-MSCs. First, increased phosphorylation of JAK2 and STAT3 in macrophages polarized by GC-MSCs was observed to be partly abrogated by either IL-6- or IL-8-specific neutralizing antibody treatment as shown in Fig. 5b. After treatment with neutralizing antibodies, both anti-IL-6 and anti-IL-8 antibodies significantly upregulated the proportion of CD86^+^ cells and reduced the proportion of CD204^+^ cells in GC-MSC-treated macrophage populations (Fig. 5c). In addition, we studied the role of JAK2/STAT3 signaling pathway in macrophage polarization by GC-MSCs, and the proportion of M2-subtype macrophages was significantly downregulated by the application of selective JAK2 and STAT3 inhibitor (JSI-124, R&D Systems, Minneapolis, MN, USA), as shown by the results of the flow cytometry (Fig. 5c). Thus, IL-6 or IL-8 secreted by GC-MSCs partly mediated their regulating effect on macrophage polarization through the activation of the JAK2/STAT3 signaling pathway. Furthermore, we investigated the contribution of IL-6, IL-8 and the JAK2/STAT3 signaling pathway to the pro-tumor effect of GC-MSC-primed macrophages. The results showed that macrophages displayed an attenuated ability to promote the migration and invasion of gastric cancer cells after co-cultured with IL-6- or IL-8-specific neutralizing antibody-pretreated GC-MSC-CM, or treated with JSI-124 before co-cultured with GC-MSC-CM (Fig. 5d, e).

### GC-MSC-primed macrophages remarkably promote the EMT process in gastric cancer cells

**Fig. 6:**
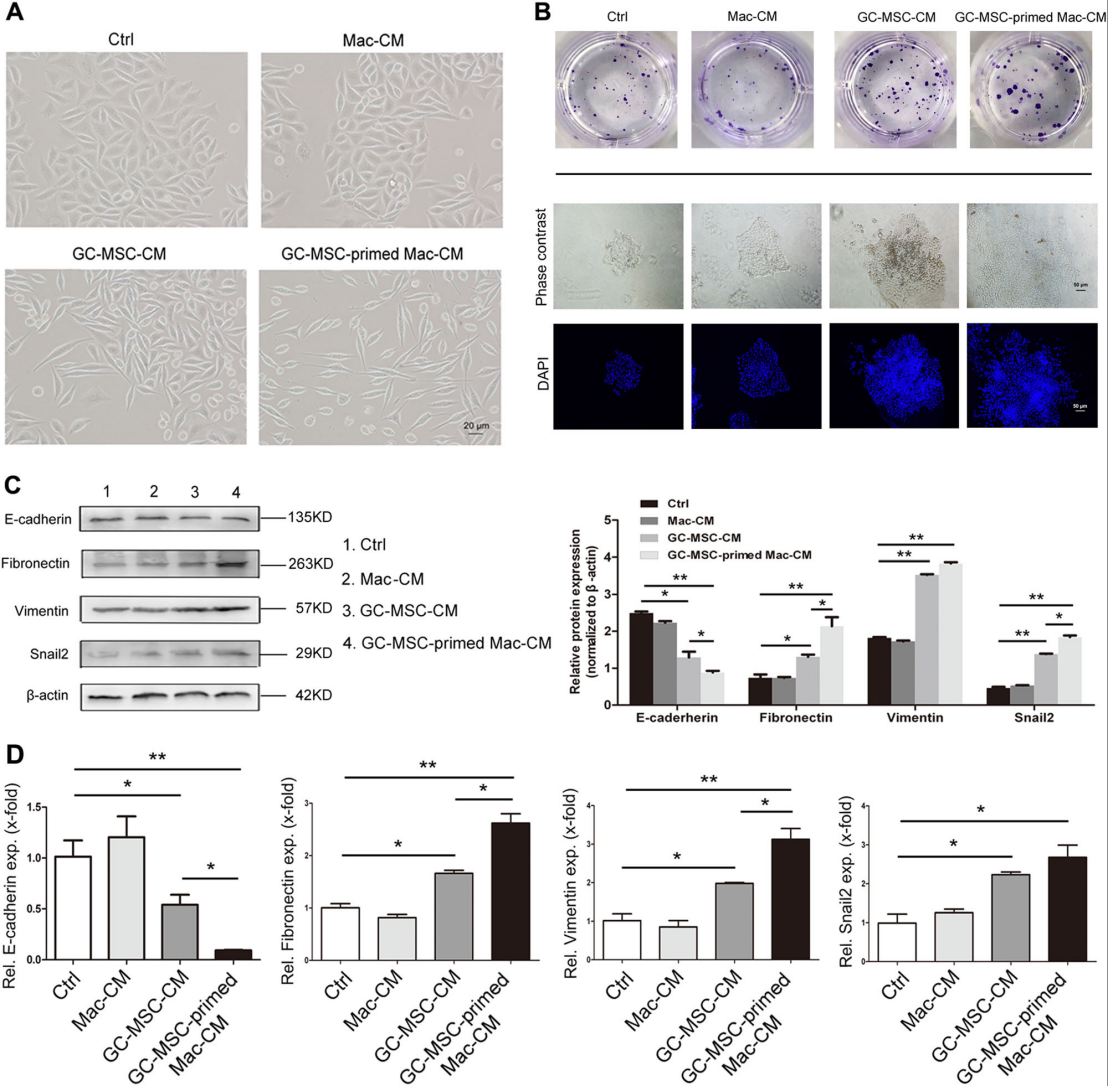
GC-MSC-primed macrophages potently advance the process of EMT in gastric caner cells. **a** Significant morphology alteration was observed in MKN-28 cells, incubated with 20% CM from macrophages, GC-MSCs and GC-MSC-primed macrophages for 5 days. The representative images were provided for each group. Scale bar, 20 µm. **b** The cloning formation of MKN-28 cells treated with 20% CM from macrophages, GC-MSCs and GC-MSC-primed macrophages for 10 days. **c** The expression levels of EMT-related markers including E-cadherin, fibronectin, vimentin and snail2 were detected by western blot in MKN-28 cells treated by 20% CM from macrophages, GC-MSCs and GC-MSC-primed macrophages for 5 days. **d** The expressions of EMT-related markers were quantified by RT-qPCR in MKN-28 cells treated by 20% CM from macrophages, GC-MSCs and GC-MSC-primed macrophages for 5 days. *P < 0.05; **P < 0.01. Ctrl: control; CM: conditioned medium; Mac: macrophages.

Previous studies have reported that macrophage-secreted cytokines can stimulate the process of epithelial mesenchymal transition (EMT), which is closely associated with cell migration^23,24^. Herein, we thoroughly studied whether promotion of gastric cancer metastasis by GC-MSC-primed macrophages is due to their promotion of the EMT process in gastric cancer cells. As shown in Fig. 6a, morphological changes in MKN-28 cells were first observed, which is one of the characteristics of EMT, after 5 days of treatment with either GC-MSC-CM or GC-MSC-primed macrophage-CM. Moreover, the scattering degree of cancer cells increased remarkably, and the cell shape was considerably more elongated in MKN-28 cells treated by GC-MSC-primed macrophage-CM compared with GC-MSC-CM; morphological changes were not obvious in MKN-28 cells treated with macrophage-CM, which were not pre-polarized by GC-MSCs (Fig. 6a). Colony formation assay revealed that MKN-28 cells treated with GC-MSC-CM and GC-MSC-primed macrophage-CM both grew faster and formed larger colonies than the control and macrophage-CM-treated groups. In GC-MSC-primed macrophage-CM-treated gastric cancer cells, mesenchymal-like cell morphology was observed considerably with a largest colony phenotype than the other groups (Fig. 6b). In addition, the expression levels of EMT-related proteins including fibronectin, vimentin and snail2 increased most significantly in MKN-28 cells treated with GC-MSC-primed macrophage-CM compared with those in any other group; the level of E-cadherin notably decreased (Fig. 6c). In agreement with this, the RT-qPCR results indicated that at 5 days of co-culture, GC-MSC-primed macrophage-CM significantly upregulated the levels of fibronectin, vimentin and snail2 mRNA in MKN-28 cells, whereas the expression level of E-cadherin was reduced. These gene alterations were all more significant than those in GC-MSC-CM -treated gastric cancer cells (Fig. 6d). Thus, our data indicate that GC-MSC-primed macrophages could potently promote the progress of EMT in gastric cancer cells, which may be responsible for their striking promotion of gastric cancer cell migration.

## Discussion

Herein, we demonstrated for the first time that macrophages play an essential role in the potent pro-tumor effect of GC-MSCs; in turn, GC-MSCs induce the polarization of macrophages into the M2 subtype through secreting IL-6 and IL-8 via the JAK2/STAT3 signaling pathway. Subsequently, these GC-MSC-primed macrophages display a prominent role in promoting the migration and invasion of gastric cancer cells via advancing the process of EMT. In total, our findings provide unique insight into the generation of pro-tumor M2-TAMs in gastric cancer. The generation of M2 macrophages ‘educated’ by GC-MSCs remarkably contributes to the re-shaping of the gastric cancer microenvironment and consequently affects the metastasis of gastric cancer.

Gastric cancer is regarded as a classic model of inflammation-related cancer, as well as one of the most common causes of cancer-related deaths worldwide^25,26^. Previously, we demonstrated that GC-MSCs play a dominant role in cancer angiogenesis and progression through the secretion of the cytokine IL-8^19^. However, the interactions between GC-MSCs and immune cells within the tumor site may contribute to tumor progression and deserve thorough investigation. Zhu et al.^27^ reported that the reciprocal interaction between GC-MSCs and neutrophils presents a novel mechanism for the role of MSCs in remodeling the cancer niche, which in turn enhances the invasiveness of gastric cancer cells and angiogenesis. Herein, we propose that macrophages are an essential factor for the pro-tumor effect of GC-MSCs and focus on the generation and function of M2 macrophages. Ren et al.^28^ reported that MSCs isolated from spontaneous lymphoma (L-MSCs) could enhance tumor growth more potently than BM-MSCs. They further identified that monocytes/macrophages, but not neutrophils, mediate the pro-tumor effect of L-MSCs, which is in support of our notion.

Macrophages, one of the major immune cells in the tumor stroma, have been demonstrated to interact with BM-MSCs in various types of tumor. Hashimoto et al.^29^ reported that BM-MSCs and PBMC-derived macrophages are recruited to tumor sites in neuroblastoma and activated to become cancer-associated fibroblasts and TAMs, respectively, which contributes to the formation of a favorable microenvironment for cancer progression. Yang et al.^30^ demonstrated that after activation by macrophages, BM-MSCs acquire a pro-inflammatory phenotype, which could promote gastric cancer cell proliferation and migration through NF-κB activation. Another study by Wolfe et al.^31^ conveyed that the interactions between BM-MSCs and macrophages induce pro-tumor properties such as enhanced migration and elevated IL-6 secretion, and macrophage-educated BM-MSCs displayed enhanced invasion and mammosphere formation.

However, urgent clarification is needed on the regulation of the phenotypes and functions of macrophages by tumor tissue-derived MSCs and the underlying mechanism, which contributes to the formation of pro-tumor TAMs. Mathew et al.^32^ identified a cell population named cancer-associated mesenchymal stem cells (CA-MSCs) in pancreatic cancer, whose ability to support tumor growth is associated with their promotion of alternative macrophage (M2) polarization. Yamada et al.^33^ also indicated that the angiogenic factor MFG-E8 drives melanoma growth through stimulating MSC-induced M2 polarization of TAMs. In the present study, we demonstrated that GC-MSCs are capable of converting the macrophages to an M2 phenotype partly mediated by IL-6 and IL-8, and these educated macrophages can markedly promote gastric cancer metastasis by advancing EMT in gastric cancer cells.

First, high expression of M2 macrophage-related proteins was detected in gastric cancer tissues, where GC-MSCs co-exist. Upon sorting cells from gastric cancer tissues by MACS technology, the TAMs displayed a significantly higher proportion of CD204^+^ cells and higher expression levels of genes indicating an M2-like immunophenotype compared to the macrophages derived from the adjacent non-cancerous tissues. Thereafter, the characterized differences in macrophages derived from human peripheral monocytes between gastric cancer patients and the healthy controls were also observed, suggesting that the phenotype of monocytes in peripheral blood was more inclined to be re-polarized to an M2 subtype in gastric cancer patients than in healthy controls.

Since the M2 subtype is the prominent phenotype of macrophages resident in GC-MSC co-existing gastric cancer tissues, we further confirmed the potential effect of GC-MSCs on macrophage polarization in an in vitro co-culture system. After co-culture for 3 days, we found that Ym-1 and Fizz-1, which are major M2 macrophage-related genes, were both highly upregulated by GC-MSC treatment of macrophages. Moreover, a high proportion of CD204^+^/CD163^+^ macrophages, as well as increasing levels of IL-6, IL-10, VEGF, and MCP-1 secretion by GC-MSC-treated macrophages, synergistically revealed a potent role of GC-MSCs in polarizing macrophages into a pro-tumor (M2) phenotype. To convey the underlying mechanism, the cytokine/chemokine profile of GC-MSCs was screened, and IL-6 and IL-8 were highly secreted by GC-MSCs compared with GCN-MSCs. Thereafter, neutralizing antibodies were used, and IL-6 and IL-8 were both confirmed as the key factors mediating the phenotype switch of macrophages by GC-MSCs. Furthermore, we also observed that GC-MSCs increased the activation of JAK2 and STAT3 in macrophages, and this effect could be partly abrogated in the presence of IL-6- or IL-8-specific neutralizing antibody, indicating that GC-MSCs promote the generation of M2 subtype TAMs by secreting IL-6 and IL-8 through regulating the JAK2/STAT3 signaling pathway.

Furthermore, the role of GC-MSC-primed macrophages in gastric cancer progression was also further assessed in our study and GC-MSC-primed macrophages facilitated the proliferation of gastric cancer cells. However, the pro-growth effect of GC-MSC-primed macrophages was less efficient than that of GC-MSCs, which has been previously identified to play a prominent promoting role in gastric cancer cell growth^19^. Hashimoto et al.^29^ found that co-culture with TAM-like macrophages did not enhance the proliferation but supported the invasive ability of tumor cells in neuroblastoma via CXCL2/CXCR2 signaling. Herein, we observed a remarkable ability of GC-MSC-primed macrophages to promote the migration and invasion of gastric cancer cells, which was even more potent than that of GC-MSCs, suggesting an important role for GC-MSC-educated TAMs in gastric cancer metastasis. These findings drew our attention and the underlying mechanism was further investigated. In addition, IL-6/IL-8-specific neutralizing antibody and selective JAK2/STAT3 inhibitor treated GC-MSC-primed macrophages were also studied and their promoting effects on the migration and invasion abilities of gastric cancer cells were partly blocked, suggesting the key roles of IL-6 and IL-8 in macrophage polarization regulated by GC-MSCs.

EMT, one of the distinctive features of tumor metastasis, is a biological process characterized by cellular and molecular changes, including the loss of cell-to-cell adhesion, acquisition of a more spindle-like morphology and upregulation of the mesenchymal proteins^34^. In this study, a marked, slender morphological change was first observed in gastric cancer cells with GC-MSC-primed macrophage treatment compared with GC-MSCs. Liu et al.^35^ noted that M2-polarized TAMs can promote EMT in pancreatic cancer cells, partially by the TLR-4/IL-10 signaling pathway. Yan et al.^36^ reported that high infiltration of TAMs is related to poor prognosis and contributes to the phenomenon of EMT in gastric cancer. Herein, our investigation demonstrated that GC-MSC-primed macrophage-CM-treated gastric cancer cells formed larger colonies and considerably mesenchymal-like cell morphology compared with the other groups. Moreover, the expression levels of vimentin, fibronectin and snail2, the three major mesenchymal genes, were all significantly upregulated in gastric cancer cells by GC-MSC-primed macrophages compared with those in the other groups. In contrast, the expression level of E-cadherin, the key epithelial-related gene, was largely reduced. Therefore, our findings indicated that GC-MSC-educated macrophages could advance the process of EMT in gastric cancer cells, which might contribute to their prominent promotion of tumor metastasis.

## Conclusion

In the present study, we highlight the unique ability of stromal GC-MSCs to induce the polarization of macrophages into a pro-tumor M2 subtype within the gastric cancer niche through the secretion of IL-6 and IL-8 via the JAK2/STAT3 signaling pathway. In turn, GC-MSC-primed macrophages remarkably promote gastric cancer metastasis by advancing the process of EMT in gastric cancer cells. Strategies that target the generation of pro-tumor TAMs in tumor tissues may provide potential therapeutic targets for gastric cancer therapy.

## Materials and methods

### Cell culture

GC-MSCs were isolated as described previously^19^. Tumor tissues were obtained from patients who underwent radical gastrectomy and the procedure was approved by the Ethics Committee of First People’s Hospital of Lianyungang (Jiangsu, China). Briefly, fresh tumor tissues were cut into 1-mm^3^-sized pieces after rinsing in antibiotics to avoid contamination and placed directly into culture dishes for 30 min to improve adhesion. Thereafter, the tissue explants were floated in a growing medium of L-DMEM (Gibco, Invitrogen Corporation, Carlsbad, CA, USA) containing 15% (v/v) fetal bovine serum (FBS; Gibco). When the spindle-like cells reached subconfluence, tissue pieces were removed and adherent cells were passaged into flasks for further expansion. The homogeneous cell population at 4–5 passages was used in this study.

Human gastric cancer cell lines BGC-823 and MKN-28 were gifts from the First Affiliated Hospital of Soochow University (Jiangsu, China) and cultured in 10% (v/v) FBS-RPMI 1640 (Gibco).

### Mouse xenograft models of gastric cancer

All animal experiments were performed in accordance with the Guidelines and Regulations for Animal Experiments of First People’s Hospital of Lianyungang. Three- to four-week-old female BALB/c nude (nu/nu) mice were purchased from the Nanjing Biomedical Research Institute of Nanjing University (Nanjing, China) and housed at the Animal Centre of Neurological Medical Institute in First People’s Hospital of Lianyungang. To establish the xenograft models, a total of 5 × 10^6^ gastric cancer cells (BGC-823 or MKN-28) in 200 µl PBS were subcutaneously injected into the right flank near the axillary fossa of the mouse. To assess the pro-tumor effect of GC-MSCs in vivo, 1 × 10^6^ cells were subcutaneously co-injected in 200 µl PBS. Animals were randomly divided into 5 mice per group and sacrificed at the end of the experiments for an observation period of 14 days. Tumor weights were recorded per mouse and tumor volumes were calculated by the modified formula of (length × width^2^)/2. Primary tumors were then collected for tissue section and homogenate preparation.

### Macrophage-depletion in the mouse xenograft model

Clodronate [dichloromethylene bisphosphonate] liposome (lipo-Cl_2_MBP) or liposomal vehicle (lipo-PBS) (Nicovan Rooijen, Amsterdam, Netherlands) was given to the mice transplanted with subcutaneous tumors every 4 days by tail vein injection at a dose of 100 μl/10 g mouse.

### Morphological analysis and immunohistochemistry

Tumor tissues collected from each group of mice were fixed in 4.0% paraformaldehyde, embedded in paraffin and cut into 4-μm sections. H&E staining was performed and used to measure the tumor burden. On the other hand, tumor sections were incubated with 3.0% hydrogen peroxide to inactivate endogenous peroxidase. After antigen retrieval, the sections were blocked in 5.0% bovine serum albumin and incubated with the following primary antibodies at 4 °C overnight: rabbit anti-mouse F4/80 (ab100790; Abcam, Cambridge, MA, USA), CD31 (ab28364, Abcam) and Ki67 (ab16667, Abcam). After washing with PBS, further incubation with a biotin-conjugated secondary antibody was performed, followed by visualization with 3,3′-diaminobenzidine (DAB) and counterstaining with hematoxylin for examination on an inverted microscope (IX71, Olympus, Tokyo, Japan).

### RNA isolation and RT-qPCR

Total RNA was extracted from tumor homogenate or cell lines using TRIzol reagent (Life Technologies, Invitrogen, Carlsbad, CA, USA) and cDNA synthesis was processed by a reverse transcription kit according to the manufacturer’s instructions (Roche Diagnostics, Indianapolis, IN, USA). qPCR was performed in a reaction mixture containing 10 μl 2 × SYBR Taq (Tli Plus) (Takara), 0.4 μl 10 μM of each primer, 0.4 μl 50 × ROX Reference Dye II (Takara), and 2 μl cDNA on an Applied Biosystems 7500 Cycler (Applied Biosystems). Primers were designed using the Primer Software produced by Invitrogen Company (Table 1). The relative expression level was determined using the 2^−ΔΔCt^ analysis method, where β-actin was used as the internal standard. All reactions were run in triplicate.

**Table 1:** Primer sequences of target genes.

Genes	Primer sequence (5′–3′)	Product	Annealing
		Size (bp)	Temperature (°C)
H-iNOS	For: TGCCACCTCCAGTCCAGTGA	129	62
	Rev: GCATCCAGCTTGACCAGAGA		
H-Ym-1	For: CCAGTGCTGCTCTGCATACA	189	62
	Rev: ATGCCGTAGAGCGTCACATC		
H-Fizz-1	For: CGTCCTCTTGCCTCCTTCTC	227	62
	Rev: ACAAGCACAGCCAGTGACAG		
H-CD163	For: ACTCTGGATCTGCTGACTTC	219	60
	Rev: CTTCCATGCTCCAGCCATTA		
H-CD204	For: AATAGTGGCAGCTCAACTCC	201	61
	Rev: ATGAGGTTGGCTTCCATGTC		
H-CD206	For: ATTAGGTGGAGAGGCAGTTG	259	62
	Rev: GTGATTCGGCATCCTGGTTG		
H-IL-10	For: GATCTCCGAGATGCCTTCAG	259	61
	Rev: ATCGATGACAGCGCCGTAGC		
H-CCL-22	For: GGCGCTTCAAGCAACTGAGG	260	65
	Rev: CAAGGCCACGGTCATCAGAG		
H-IL-23	For: CCAGAGACGCGCTGAACAGA	606	64
	Rev: TTGAAGCGGAGAAGGAGACG		
H-E-cadherin	For: GCCGAGAGCTACACGTTCAC	355	60
	Rev: GCTTTCTGAGGCCAGGAGAG		
H-fibronectin	For: GCGGACCTACCTAGGCAATG	269	62
	Rev: TCCTCCAGGTGTCACCAATC		
H-vimentin	For: CGCCAACTACATCGACAAGG	306	62
	Rev: GCCAGAGACGCATTGTCAAC		
H-snail2	For: ACTACAGCGAACTGGACACA	743	60
	Rev: ACAGCAGCCAGATTCCTCAT		
H-β-actin	For: TGGACTTCGAGCAAGAGATG	207	60
	Rev: GGATGTCCACGTCACACTTC		
M-iNOS	For: ACTGCAAGAGAACGGAGAAC	347	59
	Rev: GCCTTGTGGTGAAGAGTGTC		
M-Ym-1	For: CCAGTGCTGCTCTGCATACA	189	62
	Rev: ATGCCGTAGAGCGTCACATC		
M-β-actin	For: ACCAGTTCGCCATGGATGAC	299	60
	Rev: CACACGCAGCTCATTGTAGA		

### Western blot analysis

Tumor homogenate or cell lysate was prepared with RIPA buffer supplemented with complete protease inhibitors. Equal amounts of proteins were separated in 8% or 12% SDS-PAGE and immunoblotted with the following primary antibodies: anti-arginase-1 (MABS388, Merck-Millipore, Billerica, MA, USA), anti-CCR-2 (ab125686, Abcam), anti-PCNA (NA03, Merck-Millipore), anti-p-JAK2 (4406T, Cell Signaling Technology, Beverly, MA, USA), anti-JAK2 (3230T, Cell Signaling Technology), anti-p-STAT3 (9145T, Cell Signaling Technology), anti-STAT3 (4904T, Cell Signaling Technology), anti-fibronectin (ab32419, Abcam), anti-vimentin (#5741P, Cell Signaling Technology), anti-snail2 (#3879P, Cell Signaling Technology), and anti-β-actin (ab8226, Abcam), followed by the secondary HRP-conjugated antibodies.

### Immunofluorescence histochemistry

Tumor tissues collected from gastric cancer patients were fixed in 4.0% paraformaldehyde, embedded in paraffin and cut into 4-μm sections. Sections were incubated with 3.0% hydrogen peroxide to inactivate endogenous peroxidase and then blocked in 5.0% bovine serum albumin after antigen retrieval. To observe the relationship between GC-MSCs and TAM location within tumor, the sections were incubated at 4 °C overnight with primary antibodies against α-SMA (NBP2–33006, Novus Biologicals, Briarwood Avenue, CO, USA) and CD204 (bs136214, absin, Shanghai, China). Subsequently, incubation with Alexa Fluor 488-labelled goat anti-mouse IgG (H + L) and Alexa Fluor 555-labelled donkey anti-rabbit IgG (H + L) (Beyotime, Shanghai, China) was performed for 1 h at 37 °C in the dark. Nuclei were counterstained with DAPI (Beyotime). Fluorescent images were acquired by a confocal laser-scanning microscope (T*i*2-E-A1, Nikon, Tokyo, Japan).

### Selection of human gastric cancer-derived TAMs

Gastric cancer and adjacent non-cancerous tissues were obtained from patients who underwent radical gastrectomy. After rinsing in antibiotics, the primary tumor tissues were dissociated into single-cell suspensions by a human tumor dissociation kit (130-095-929, Miltenyi Biotech, Cologne, Germany) according to the manufacturer’s instructions on a gentleMACS™ Octo Dissociator with Heaters (Miltenyi Biotech). Thereafter, TAMs were obtained by performing positive selection with magnetically labeled human CD14 beads (Miltenyi Biotech) according to the manufacturer’s instructions.

### Preparation of human peripheral monocyte-derived macrophages

First, peripheral blood mononuclear cells (PBMCs) were isolated by Ficoll-Hypaque (1.077 g) (LTS1077, tbdscience, Tianjin, China). Then, CD14^+^ cells were isolated from the PBMCs by performing CD14 bead positive selection (Miltenyi Biotech) according to the manufacturer’s instructions. After confirming the purity of CD14^+^ monocytes by flow cytometry, the cells were cultured in 6-well plates at 5 × 10^5^ cells/ml in 10% FBS-RPMI 1640 and differentiated into macrophages by incubation with 50 ng/ml phorbol 12-myristate 13-acetate (PMA) for 24 h.

### Flow cytometry

In this study, flow cytometric analysis was used to detect the immunophenotype of macrophages in gastric cancer tissues, peripheral blood or a co-culture system with GC-MSCs. Briefly, a single-cell suspension of macrophages was first incubated with PE-CD204 (FAB2708P, R&D Systems), APC-CD163 (ab134416, Abcam) or APC-CD86 (560956, BD Pharmingen, San Jose, CA, USA) for 30 min at 4 °C in the dark. After washing, the labeled cells were resuspended in PBS and analyzed on a FACSCanto II flow cytometer (BD Biosciences, Sparks, MD, USA). As negative controls, isotype-matched antibodies with the corresponding fluorescent labels were used.

### Luminex immunoassay

In a co-culture system with GC-MSCs, the cytokine profile of macrophages was analyzed by Luminex assay. After 3 days of co-culture, the supernatant of cell cultivation media was collected so that the concentration and culture time were coincident to make the data comparable. Then, the levels of IL-6, IL-10, tumor necrosis factor-α (TNF-α), IFN-γ-induced protein 10 (IP-10), C-C motif chemokine ligand 5 (CCL5/RANTES), vascular endothelial growth factor (VEGF), macrophage inflammatory protein-1α (MIP-1α) and monocyte chemoattractant protein-1 (MCP-1) were quantified simultaneously using a human cytokine/chemokine kit (MPXHCYTO-60K, Merck-Millipore) on a multiplexed bead array immunoassay platform (Bio-Plex 200, Bio-Rad Laboratories, Hercules, CA, USA). All reactions were run in triplicate. The differences in cytokine profile between GC-MSCs and MSCs from adjacent non-cancerous gastric cancer tissues (GCN-MSCs) were also analyzed by Luminex immunoassay.

### MTT assay

In 96-well plates, MKN-28 cells (2 × 10^4^ cells/ml) were incubated with 20% CM from macrophages, GC-MSCs or GC-MSC-primed macrophages, respectively. At 3 or 5 days, MTT solution (5 mg/ml) was added into each well and incubated with the cells at 37 °C for 4 h. Then, the solution was discarded and 150 μl DMSO was added into the remaining cells for dissolving formazan crystals. The optical density (OD) at 490 nm was measured by a microplate reader (Bio-Rad Laboratories).

### Cell migration and invasion analysis

To examine the effects of GC-MSC-primed macrophages on gastric cancer cell migration and invasion, cell assays were performed in a 24-well transwell chamber. For the migration assay, MKN-28 cells were seeded in serum-free RPMI-1640 at a density of 2 × 10^5^ cells/ml in the upper chamber of transwell inserts. For cell invasion assays, cells at 3 × 10^5^ cells/ml were plated in the upper compartment with Matrigel-coated membranes (Corning Life Sciences, Bedford, MA, USA). Twenty percent CM from macrophages, GC-MSCs or GC-MSC-primed macrophages was added into the lower chamber. After incubation at 37 °C for 24 h, the inserts were discarded and the upper side of the filter was swabbed to remove the non-migratory cells. After fixation in 4% paraformaldehyde, the filters were stained with 0.5% crystal violet for 20 min. The stained migrated or invaded cells were visualized and photographed by an inverted microscope (IX71), and 8 randomly non-overlapping high-power fields (HPFs, × 200) were selected for counting the migrated cells.

### Neutralization assay by IL-6/IL-8 blockade

To evaluate the effect of IL-6 or IL-8 on M2-like macrophage polarization prompted by GC-MSCs, a neutralization assay was performed in our study. Briefly, macrophages were treated with 20% GC-MSC-CM alone or together with a human IL-6 neutralizing antibody (554541, BD Pharmingen) at a final concentration of 2 ng/ml, or with a human IL-8 neutralizing antibody (554726, BD Pharmingen) at a final concentration of 150 μg/ml, or with a mixture of IL-6 and IL-8 neutralizing antibodies. Mixtures of GC-MSC-CM and neutralizing antibodies were incubated at 4 °C overnight before being added to macrophages. The immunophenotype and macrophage polarization-related signaling pathway were assessed after neutralizing antibody treatment.

### Colony formation assay

The colony forming ability of MKN-28 cells was determined in duplicate at 200 cells/well in a 24-well plate. The tumor cells were incubated in 20% CM from macrophages, GC-MSCs or GC-MSC-primed macrophages, respectively, at 37 °C in a humidified incubator. After 10 days, the adherent cells were washed with PBS, fixed with 4% paraformaldehyde and stained with 0.5% Crystal violet or DAPI.

### Statistical analysis

The results are expressed as the mean ± SEM. Data were analyzed with GraphPad Prism software 6.0 (GraphPad Software, La Jolla, CA, USA). Statistical analysis was performed by non-parametric Mann-Whitney *U* test with SPSS 16.0 statistical software. *P* < 0.05 was considered statistically significant.

## Supplementary information

Supplementary Figure 1

Supplementary Figure 2

Supplementary Figure 3

Supplemental figure legends
