# Supplementary figures and images for: Gastric cancer-derived mesenchymal stromal cells trigger M2 macrophage polarization that promotes metastasis and EMT in gastric cancer
Source: Cell Death Dis. 2019 Dec 4;10(12):918. doi: 10.1038/s41419-019-2131-y (PMC6892854; doi:10.1038/s41419-019-2131-y)

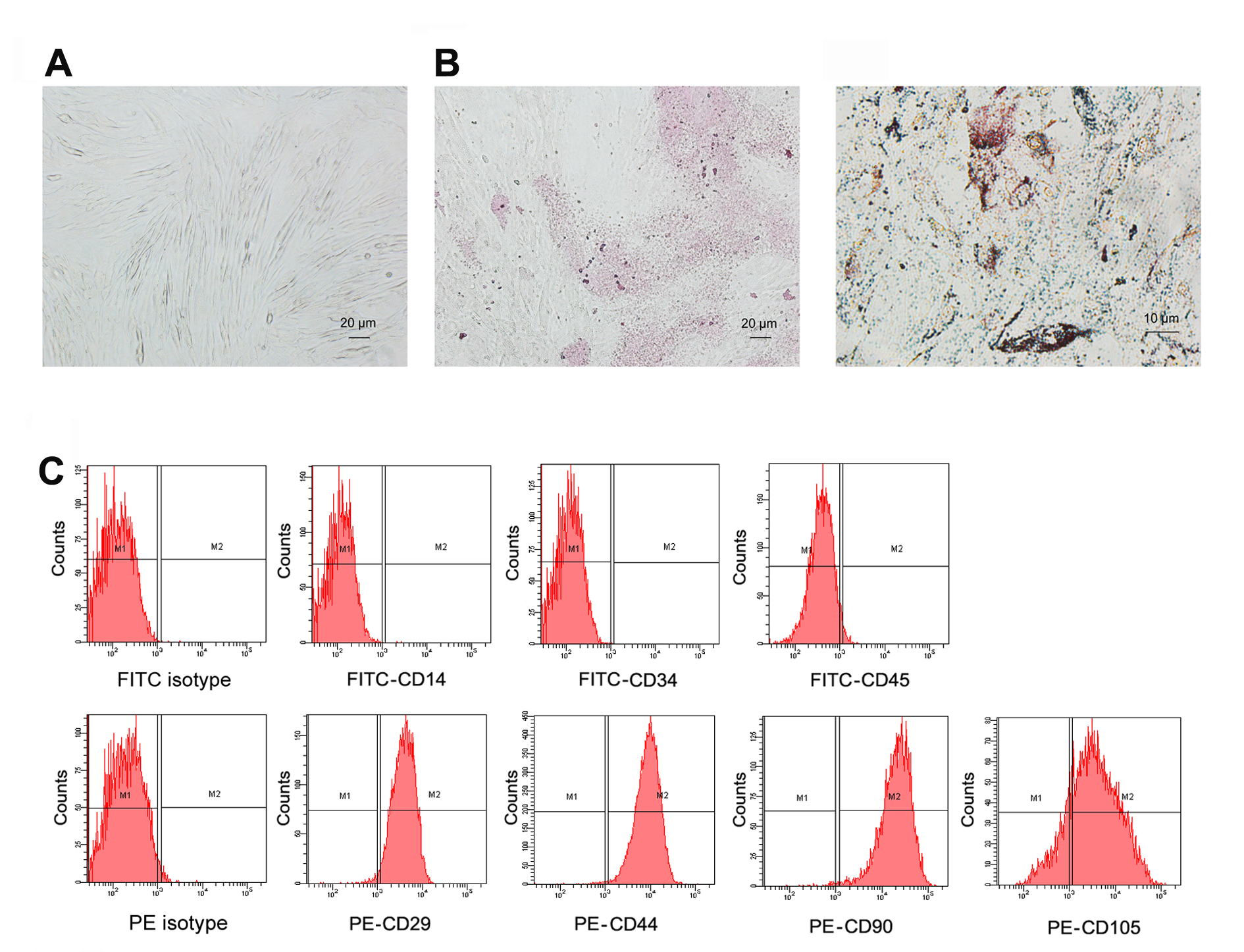

Supplement: Supplementary file 1 — Supplementary Figure 1 [file 41419_2019_2131_MOESM1_ESM.tif]

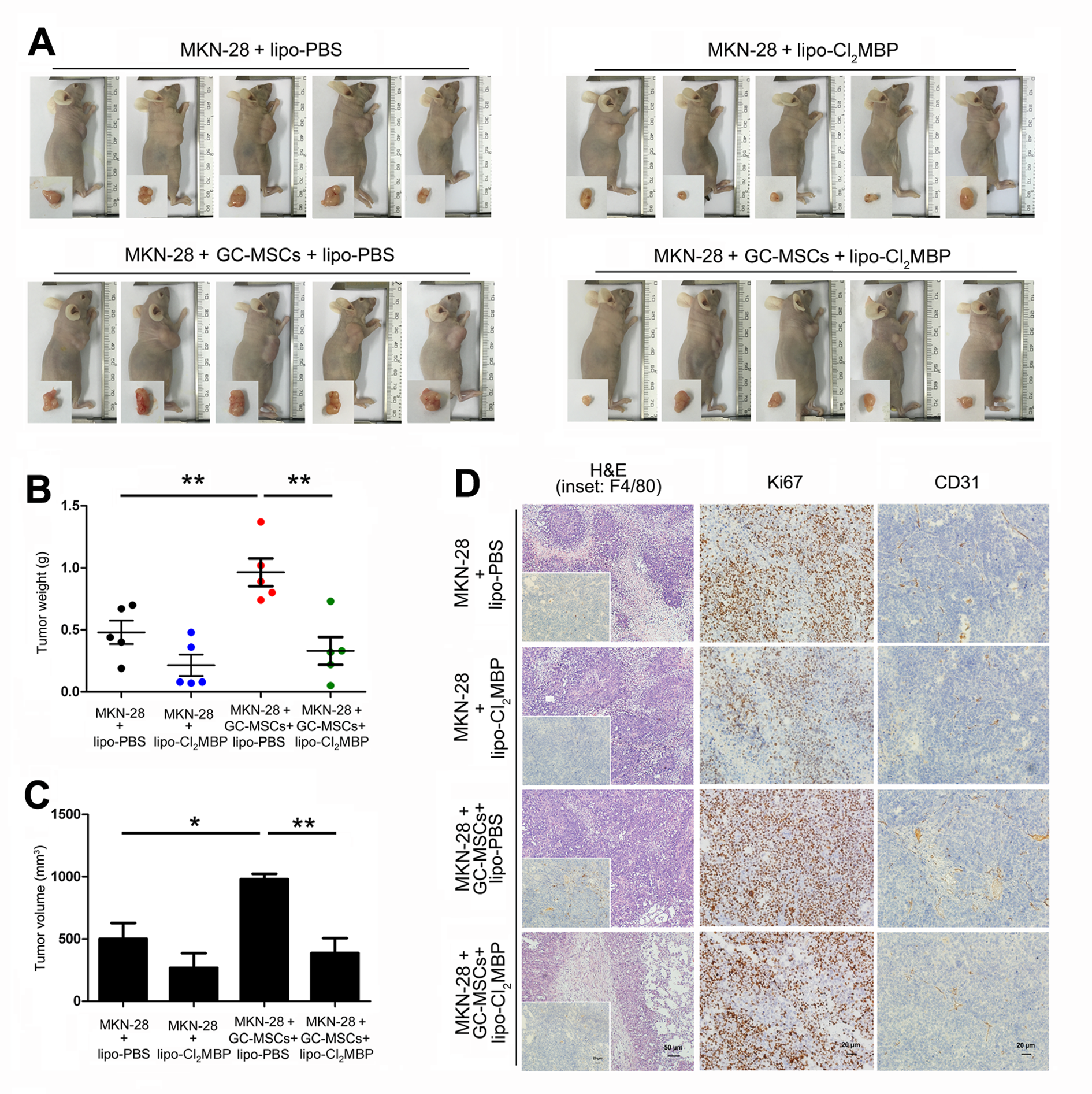

Supplement: Supplementary file 2 — Supplementary Figure 2 [file 41419_2019_2131_MOESM2_ESM.tif]

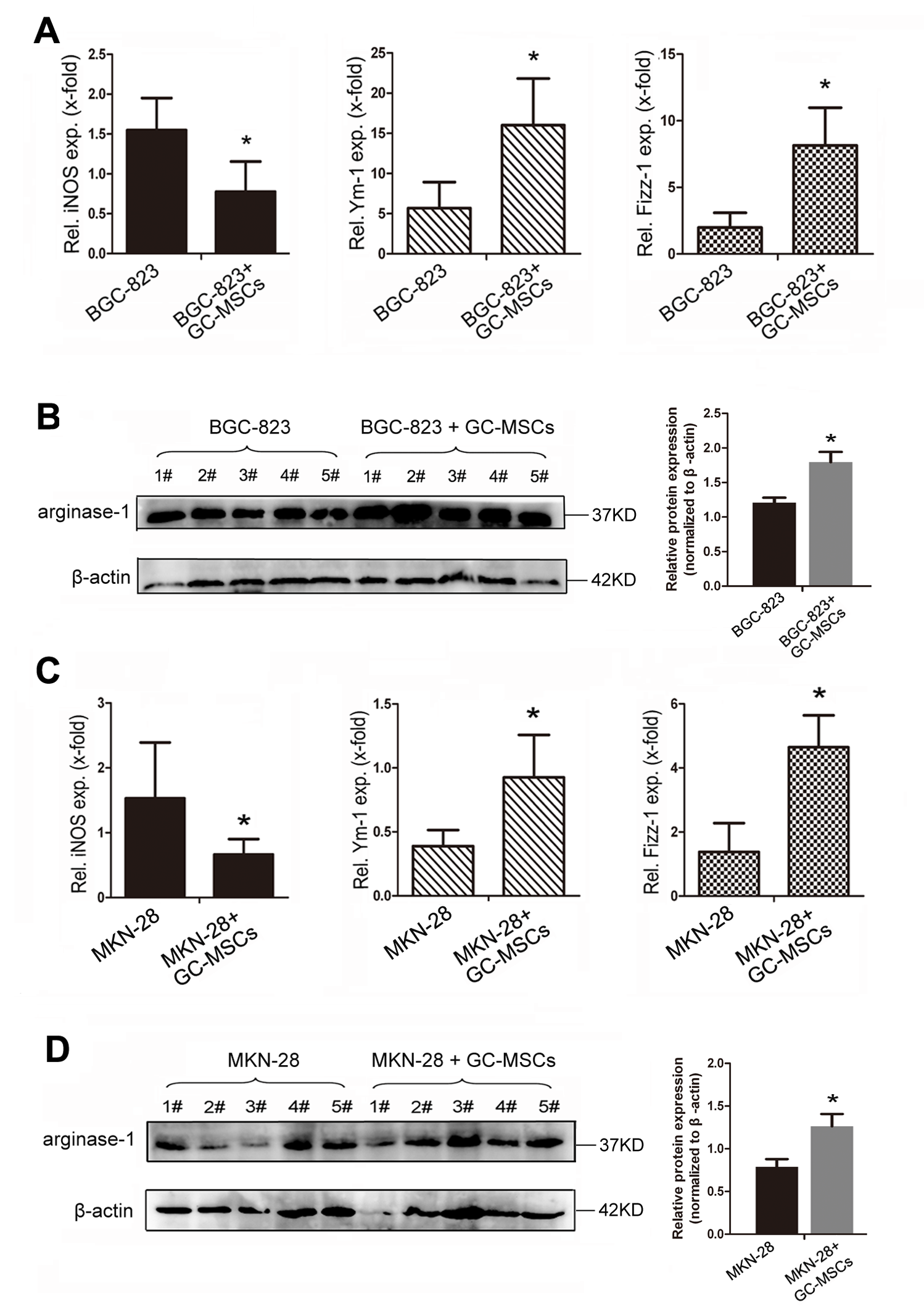

Supplement: Supplementary file 3 — Supplementary Figure 3 [file 41419_2019_2131_MOESM3_ESM.tif]
