# Supplementary material for: Gastric cancer-derived mesenchymal stromal cells trigger M2 macrophage polarization that promotes metastasis and EMT in gastric cancer
Source: Cell Death Dis. 2019 Dec 4;10(12):918. doi: 10.1038/s41419-019-2131-y (PMC6892854; doi:10.1038/s41419-019-2131-y)
Supplement: Supplementary file 4 — Supplemental figure legends [file 41419_2019_2131_MOESM4_ESM.doc]

**Supplementary Fig. 1 Characterization of GC-MSCs isolated from tumor tissues of gastric cancer patients. a** Representative photograph of spindle-shaped GC-MSCs at passage 4. Scale bar, 20 µm. **b** Representative images of GC-MSCs differentiated into mineralizing cells with alizarin red S staining (left; scale bar, 20 µm) and adipogenic cells with Oil red O staining (right; scale bar, 10 µm). **c** Immunophenotype of GC-MSCs was analyzed by flow cytometry.

**Supplementary Fig. 2 Macrophages play an essential role in the pro-tumor effect of GC-MSCs in MKN-28-bearing mice.** **a** Gross morphology of tumor at 14 days after MKN-28 transplantation with distinct treatments. **b** Tumor weight and **c** volume evaluated in each group of mice at 14 days after MKN-28 transplantation with distinct treatments. *n* = 5 in each group. **d** Representative histopathology images of tumors collected from mice co-injected by MKN-28 and GC-MSCs, with or without macrophage-depletion. H&E staining: scale bar, 50 µm; Immunohistochemistry for F4/80 (inset): scale bar, 20 µm; for Ki67: scale bar, 20 µm; for CD31: scale bar, 20 µm. **P*<0.05; ***P*<0.01.

**Supplementary Fig. 3 M2-like macrophage-related markers are significantly up-regulated in mouse tumor tissues co-injected with GC-MSCs.** **a** Expression levels of iNOS, Ym-1 and Fizz-1 mRNA in tumor tissues were analyzed by RT-qPCR at 14 days after BGC-823 transplantation, with or without GC-MSC co-injection. **b** The expression of arginase-1 in mouse tumors was detected by western blot at 14 days after BGC-823 transplantation, with or without GC-MSC co-injection. **c** Expression levels of iNOS, Ym-1 and Fizz-1 mRNA in tumor tissues were analyzed by RT-qPCR at 14 days after MKN-28 transplantation, with or without GC-MSC co-injection. **d** The expressions of arginase-1 in mouse tumors was detected by western blot at 14 days after MKN-28 transplantation, with or without GC-MSC co-injection. *n* = 5 in each group. **P*<0.05.
